# Case 4 – A 59-Year-Old Woman with Rheumatic Mitral Valve Disease (Severe Stenosis and Regurgitation), Severe Dyspnea, Shock and Pulmonary Condensation

**DOI:** 10.5935/abc.20180157

**Published:** 2018-08

**Authors:** Desiderio Favarato, Vera Demarchi Aiello

**Affiliations:** Instituto do Coração (InCor) HC-FMUSP, São Paulo, SP - Brazil

**Keywords:** Mitral Valve/complications, Heart Murmurs, Mitral Valve Insufficiency, Arrhythmias, Cardiac, Pulmonary Embolism

A 59-year-old female patient with double mitral lesion was hospitalized with fever, cough and worsening dyspnea with shock.

At 58 years old, the patient reported onset of dyspnea in medium exertions for five months, associated with dry cough at night with dyspnea, which was relieved with orthostatism. Cardiac murmur was detected and the patient was referred to InCor, a heart specialist hospital, for treatment (17/Sept/2010).

There was no reference to rheumatic outbreaks in the past, and the patient had arterial hypertension and hypothyroidism.

Physical exam when the patient was first examined (17/Sep/2010) showed the patient weighed 73 Kg, was 1.55 m tall, body mass index was 30.6 kg/m^2^, cardiac frequency was 88 bpm, arterial blood pressure 140 x 90 mmHg; pulmonary auscultation resulted normal; cardiac auscultation revealed hypophonic 1^st^ heart sound, hypophonic pulmonary component of 2^nd^ heart sound and mitral holosystolic murmur ++++/6+; abdomen exam resulted normal; there was no edema in the lower limbs and pulse palpation was normal.

The ECG (14/Sep/2010) showed sinus tachycardia, with 127 bpm frequency, PR interval 200 ms, dQRS 92 ms, SÂQRS + 150º reverse, QTc 459 ms, overload of the left atrium and indirect signs of overload of the right atrium (Peñaloza-Tranchesi signal), low-voltage front plane and overload of the right ventricle (Figure 1).

**Figure 1:**
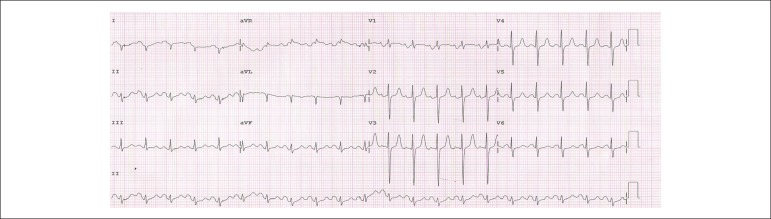
ECG. Sinus tachycardia. Overloaded left atrium and indirect signs of overloaded right atrium (Peñaloza-Tranchesi signal) and overloaded right ventricle.

Laboratorial exams (14/Sep/2010) showed red blood cells 5.0 million/mm^3^, hemoglobin 14.6 g/dL, hematocrit 45%, creatinine 1.08 mg/dL (FG = 55L/min/1.73 m^2^), potassium 4.4 mEq/L and sodium 142 mEq/L.

The echocardiogram (25/Aug/2010) revealed aortic diameter 25 mm, left atrium 52 mm, right ventricle 44 mm, left ventricle 34/21 mm, ejection fraction 70%, septum thickness and posterior wall 11 mm; there was no alteration in segment contraction of the left ventricle; right ventricle’s systolic function was normal; mitral valve presented thickened cusps with commissural fusion and reduced opening, compatible with a severely compromised rheumatic condition, and there was significant valve insufficiency. The maximum diastolic gradient between the left atrium and the ventricle was estimated at 30 mm Hg, and the medium, at 18 mm Hg; the aortic valve showed discrete signs of fibrocalcification without functional alterations; the tricuspid valve had severe insufficiency. Pulmonary artery systolic pressure was estimated at 140 mmHg.

Losartan 100 mg, furosemide 40 mg, digoxin 0,25 mg and acetylsalicylic acid 100 mg daily were prescribed.

Surgical treatment of the mitral valve was indicated.

In December 2010 the patient sought emergency medical attention due to tachycardia and dyspnea.

The ECG (16/Dec/2010) revealed nodal reentrant tachycardia, with 178 bpm frequency (Figure 2). The patient underwent chemical cardioversion with intravenous amiodarone.

**Figure 2:**
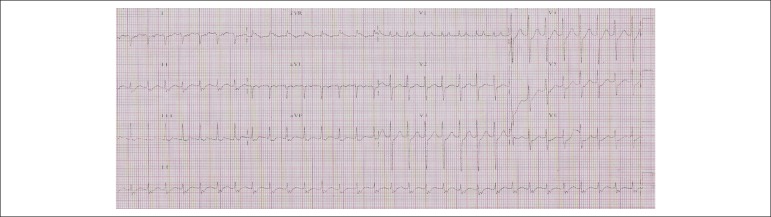
ECG. Nodal reentrant tachycardia, retrograde P wave (negative II, III and aVF) after QRS with short PR interval (< 70 ms)

At the outpatient ward (5/Apr/2011) the patient was asymptomatic, with controlled blood pressure (120/80 mmHg) and heart rate of 84 bpm, and the physical exam resulted normal, except for preexisting alterations in the cardiac auscultation. The patient used 200 mg of amiodarone, 40 mg of furosemide, 100 mg of losartan and 60 mg of diltiazem.

The patient continued waiting to be operated and on 16/Sep/2011 she sought emergency medical care, with dyspnea in small exertions and productive cough with purulent sputum, and no fever was reported.

The physical exam showed a sleepy patient, with cold extremities and a heart rate of 98 bpm, blood pressure 93 x 58 mmHg. Pulmonary auscultation revealed crackling rales in the lower third of both hemithorax; cardiac auscultation revealed rhythmic heart sounds, mitral regurgitation systolic murmur +++/6+ and diastolic arrhythmia ++/6+; the abdomen had no abnormalities and there was edema ++/4+ in the lower limbs.

The ECG (16/Sep/2011) showed sinus rhythm with 97 bpm frequency, PR 168 ms, dQRS 89 ms, SÂQRS + 150º reverse, QTc 513 ms, biatrial overload, giant P wave positive at V1 and right ventricular overload (Figure 3).

**Figure 3:**
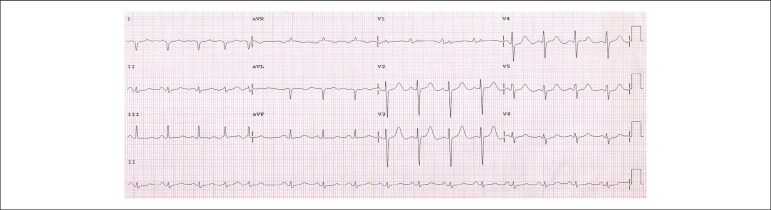
ECG. Sinus rhythm; Both atria overloaded with giant P wave positive at V_1_ and overloaded right ventricle.

The echocardiogram (17/Sep/2011) revealed hypokinesia of the right and left ventricles, the latter with 55% ejection fraction, from moderate to strong mitral insufficiency, maximal mitral transvalve gradient at 22 mm Hg and medium, at 13 mmHg. Pulmonary artery pressure was estimated at 81 mmHg; however, the patient had systemic arterial hypotension, 53 mmHg medium pressure.

Antibiotics were prescribed (ceftriaxone and clarithromycin), which later were changed for the piperacillin/tazobactam and vancomycin association, in addition to vasoactive amines, oxygen mask, and then orotracheal intubation for respiratory support.

Laboratorial exams (17/Sep/2011) showed: red blood cells 4.2 million/mm^3^, hemoglobin 12 g/dL, hematocrit 39%, VCM 93 fL, RDW-CV 17.9%, leukocytes 13840/mm^3^ (90% neutrophils, 7% lymphocytes and 3% monocytes), platelets 161000/mm^3^, urea 63m/dL, creatinine 1.44 m/dL (FG = 40 mL/min/1.73 m^2^), magnesium 1.3 mEq/L, sodium 137 mEq/L, potassium 3.9 mEq/L, prothrombin time (INR) 1.7 and APTT rel 1.26.

Thorax radiography (18/Sep/2011) revealed pulmonary congestion, opacification at the right base and increased cardiac area (presence of double contour and bulging unfolding of the mid aortic arch) (Figure 4).

**Figure 4:**
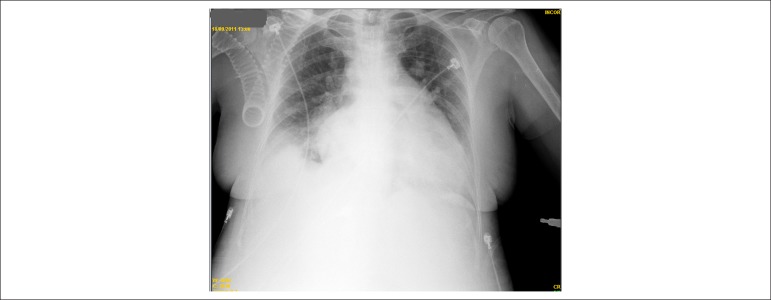
Chest X-ray. Pulmonary congestion, opacification at the right base, and cardiomegaly with double contour (enlargement of the left atrium), and abnormal enlargement or bulging at mid-arch of the aorta (enlargement of the right ventricle).

The coronary angiography (20/Sep/2011) did not reveal coronary obstructions; and there was severe calcification of the mitral valve.

Laboratorial exams (20/Sep/2011) revealed hemoglobin 11 g/dL, hematocrit 36%, VCM 92 fL, RDW-CV 17.8%, leukocytes 13440/mm^3^ (90% neutrophils, 7% lymphocytes, 3% monocytes), platelets 142000/mm^3^, urea 74 mg/dL, creatinine 1.97 mg/dL (FG = 28 mL/min/1.73 m^2^), AST 34 U/L, ALT 34 U/L, calcium 4 mEq/L, magnesium 1.3 mEq/L, arterial lactate 155 m dL.

Exams of 21/Sep/2011 showed hemoglobin 10.5 g/dL, hematocrit 37%, VCM 102 fL, RDW-CV 16.8%, leukocytes 17840 mm/mm^3^ (93% neutrophils, 5% lymphocytes, 2% monocytes), platelets 148000/mm^3^, urea 104 mg/dL, creatinine 2.88 mg/dl (FG = 18 mL/min/1.73 m^2^), sodium 145 mEq/L, potassium 4.5 mEq/L, AST 1863 U/L, ALT 426 U/L, gammaGT 87 U/L, alkaline phosphatase 152 U/L, magnesium 1.9 mE L, total bilirubin 4.05 mg/dL, direct bilirubin 3.5 mg/dL, arterial lactate 173 mg/dL; arterial gasometry: pH 7, 16, pCO_2_ 32.7 mm Hg, pO_2_ 160 mm Hg, O_2_ Saturation 99%, bicarbonate 11mEq/L, base excess (-) 16.2 mEq/L; TP (INR) 3.2; TTPA rel times 3.13.

Thorax radiography (21/Sep/2011) showed hypotransparency in the right pulmonary base and an increase in the cardiac area (presence of double contour and abnormal enlargement or bulging at mid-arch of the aorta) (Figure 5).

**Figure 5:**
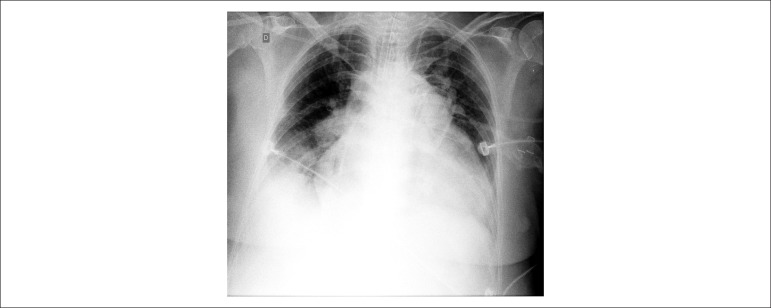
Chest X-ray. More penetrated than the previous one. Presence of endotracheal cannula. Pulmonary congestion, opacification at the right base and cardiomegaly with double contour (enlargement of the left atrium) and abnormal enlargement or bulging at mid-arch of the aorta (right ventricle enlargement).

During hospitalization the patient developed hemodynamic instability, consumption coagulopathy, and presented cardiac arrest in pulseless electrical activity, with no response to resuscitation maneuvers, and she passed away (16h and 55min; 21/Sep/2011).

## Clinical aspects

This is about a 59-year-old woman with double mitral lesions with pulmonary hypertension who, while awaiting surgery, had arterial hypotension, respiratory failure with hypotransparency in the right lung field. It evolved without improvement with vasoactive drugs, orotracheal intubation and antibiotic therapy, and she passed away in electrical activity without pulse.

The etiology of this patient's valve disease should be attributed to rheumatic fever, although there is no description of an acute outbreak of that disease in the patient’s medical history. That is not unusual in a rheumatic disease scenario once using echocardiogram raises its frequency from 5 to 10 times when compared to a clinical diagnosis.^1^^-^^4^

That difference in frequency between clinical diagnosis and echocardiography may be due to the autoimmune response triggered by molecular mimicry,^5^ which may go ahead with predominance of humoral response mediated by Th2 lymphocytes, causing more symptoms and leading more easily to the use of secondary prophylaxis. Others show a predominance of cellular response, mediated by Th1 lymphocytes with milder forms of clinical manifestations, but those where cardiac involvement predominates. Thus, patients who would benefit the most from the use of that prophylaxis fail to do it and are subject to relapses that aggravate valve lesions.^6^

Those subtypes of CD4 + (Th1 and Th2) lymphocytes produce different cytokines, those of Th1-type produce interleukin-2 and interferon-gamma cytokines, and those of Th2 subtype secrete Interleukins 4, 5 and 10.^7^^-^^9^

The guidelines of the World Health Organization and the US National Institute of Health (NIH) have defined the diagnosis of rheumatic heart disease by the presence of cardiac murmur consistent with mitral or aortic insufficiency, and echocardiographic evidence of rheumatic valve damage, or history of acute rheumatic fever without echocardiogram done in the acute outbreak.^10^

The predominant clinical onset of the heart rheumatic disease is dyspnea, which occurs between the third and fourth decade in life, mostly in women.^11^

In this case, clinical manifestations happened later, but like in the rheumatic disease, they presented alterations in the mitral valve, the valve most frequently affected in the disease. Mitral regurgitation usually occurs earlier than stenosis, attributed to persistent or recurrent valvulitis.^12^

Despite its earlier onset, mitral regurgitation generally has a longer asymptomatic period due to increased atrial compliance due to its progressive increase, maintenance of cardiac output by dilatation of the left ventricle, and decreased regurgitation fraction in exertions to decrease peripheral resistance in the exertion.

Still on this case, the initial clinical scenario of de-compensation due to the presence of tachycardia, once a shorter diastole is more detrimental to mitral stenosis, suggests its predominance. Additionally, the first echocardiogram (2010) was more compatible with the predominance of mitral stenosis with major hemodynamic repercussion, once the dimensions of the left ventricle were normal and there was an increase in the left atrium and in the pulmonary systolic pressure, as well as dilation of the right ventricle.

Echocardiographic criteria for severe stenosis were present, although the valve area was not calculated, which would be less than 1.0 cm^2^/m^2^ or < 1.5 cm^2^, there was fusion of the cusps, in addition to the pressure gradient between the left atrium and the upper ventricle at 10 mmHg, and pulmonary arterial hypertension above 50 mmHg.^13^^,^^14^

The indication for percutaneous and/or surgical treatment is based on the presence of symptoms or atrial fibrillation, or systemic embolism, in patients using anticoagulant and moderate or severe mitral stenosis, and in asymptomatic patients with pulmonary systolic pressure ≥ 80 mmHg. Percutaneous valvuloplasty still requires the presence of favorable morphology - mobile thin cusps, free from calcification.

 In the second echocardiogram, one year after the accompaniment had started, the predominance of mitral insufficiency was not discarded because there was malfunction of the left ventricle.

The right time of an indication for surgery in patients with severe mitral incompetence has always been controversial due to the symptoms’ late onset. Surgical treatment is indicated for the onset of symptoms, of dilatation (systolic diameter > 4.5 cm), or left ventricular dysfunction (ejection fraction < 60%). The guidelines define severe mitral regurgitation based on several parameters: valve morphology - ring dilation ≥ 3.5 cm; characteristics of the regurgitation jet - a rotating jet (multicolor) that reaches the posterior atrial wall, or lateral jet that fills at least 40% of the atrial surface; the *vena contracta* - width of the jet next to the valve ≥ 0.7 cm; effective regurgitation orifice ≥ 0.4 cm^2^; regurgitation volume ≥ 60 mL; regurgitation fraction ≥ 50%, ventricular filling pattern – ratio of the mitral valve’s velocity-time integral and the aortic valve above 1.3; wave velocity E ≥ 150 cm/s; pulmonary artery systolic pressure ≥ 50 mm Hg; indexed atrial volume 60 mL/m^2^; and left ventricle systolic diameter > 4.5 cm.^3^^,^^15^

Thus, whichever was the predominance of valvular dysfunction, the patient already had indication for surgical treatment of the mitral valve because there was already severe hemodynamic repercussion and, as it usually happens in valvopathy with a rheumatic origin, there was a double lesion.

Other ways in which the disease may appear are atrial arrhythmias, the most common being atrial fibrillation, embolic events, acute heart failure, or infective endocarditis.

In this case there was de-compensation of heart failure due to nodal reentrant tachycardia, arrhythmia is usually not associated with mitral valvopathy. In mitral stenosis there is usually atrial fibrillation due to dilation and atrial fibrosis, in addition to an inflammatory process in the acute phase (Aschoff nodules).

Atrial phenomena in mitral insufficiency are similar to those of stenosis as interstitial fribrosis and inflammation; however, there is no hypertrophy, myolysis and necrosis of atrial myocytes.^16^

As to the patient's final condition, there are three possible causes: infectious endocarditis, pulmonary infection or pulmonary thromboembolism.

For the diagnosis of endocarditis there would be only fever and de-compensation of heart failure, lacking worsened murmur and vegetation in the valves.

In other words, the involvement of the endocardium in systemic infection has not been proven. Blood culture is not a diagnostic criterion. The clinical criteria of strong suspicion are: new valve injury (insufficiency), embolic events of unknown origin, sepsis of unknown origin, hematuria and fever in a patient who has a prosthesis, previous valvopathy and dental or endoscopic manipulation of the colons, new conduction disorders (atrioventricular blockage due to perivalvular abscess), first episode of cardiac de-compensation, positive blood cultures, skin complications (Osler spots, Janeway) or ophthalmic complications (Roth), peripheral abscesses (kidneys, spleen).^17^

According to their frequency, the most common complications - heart failure > systemic embolization > stroke > intracardiac abscess.^18^

However, endocarditis cannot be ruled out because in rheumatic valvular disease it can be difficult to diagnose the vegetation.

Infection at any place can be responsible for the de-compensation of heart failure in patients with severe valvopathy. In this case, pneumonia was suspected due to the presence of suggestive image at the right base (Figure 4) and leukocytosis, and antibiotic therapy was introduced; however, there was no change in the clinical situation compatible with the presence of pneumonia.

As the last and most probable cause of the onset of the patient's final hemodynamic status there is pulmonary thromboembolism.

The clinical situation is very non-specific and may be mixed up with acute coronary syndrome or pneumonia. Favoring it there is the image at the base of the right lung and dysfunction of the right ventricle.

In the “International Cooperative Pulmonary Embolism Registry (ICOPER)” pulmonary thromboembolism is associated with the presence of heart failure (hazard ratio 2.4), right ventricle hypokinesia (2.0), systolic arterial hypotension < 90 mmHg (2.9), age > 70 years (1.6), cancer (2.3), chronic obstructive pulmonary disease (1.8). In the same sense, pulmonary thromboembolism with right ventricle hypokinesia doubled the mortality within 3 months.^19^

The exam deemed "gold standard" in the diagnosis of pulmonary thromboembolism is angiotomography, but failing that, or due to the patients’ hemodynamic instability, the finding of dilation and right ventricle dysfunction on the echocardiogram can be diagnostic alternatives.

And as to hepatic alterations – elevation of transaminases and disorders in coagulation (elevation of TAP-INR- and relation of APTT times) – they are compatible with ischemic hepatitis with extensive liver necrosis due to low cardiac output in patients with high ventricular diastolic pressures. Its onset takes on average one week after the episode of low hepatic flow leading to centrilobular necrosis.^20^ (**Dr. Desiderio Favarato**)

**Diagnostic hypotheses:** Rheumatic mitral valvopathy (double lesion), pulmonary thromboembolism, cardiogenic shock, multiple organ failure. (**Dr. Desiderio Favarato**)

## Necropsy

**Figure 6:**
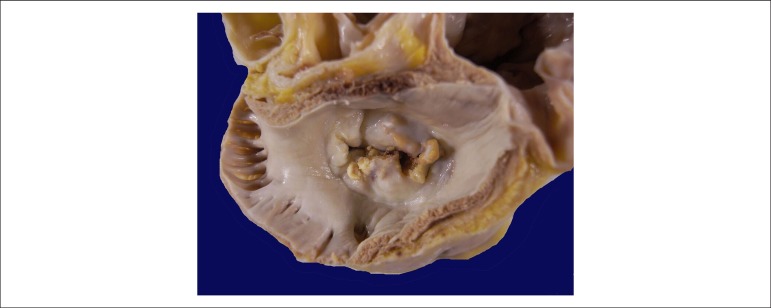
Right atrium open showing the mitral valve with fusion of commissures and multiple foci of calcification.

**Figure 7:**
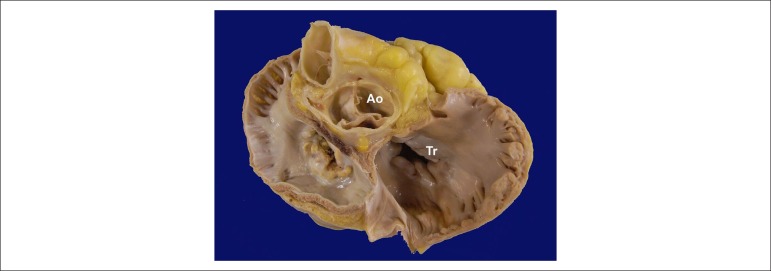
Base of the heart from where the atria were removed. Observe that the aortic valve (Ao) is preserved and the tricuspid valve (Tr) shows insufficiency secondary to the ring dilatation.

**Figure 8:**
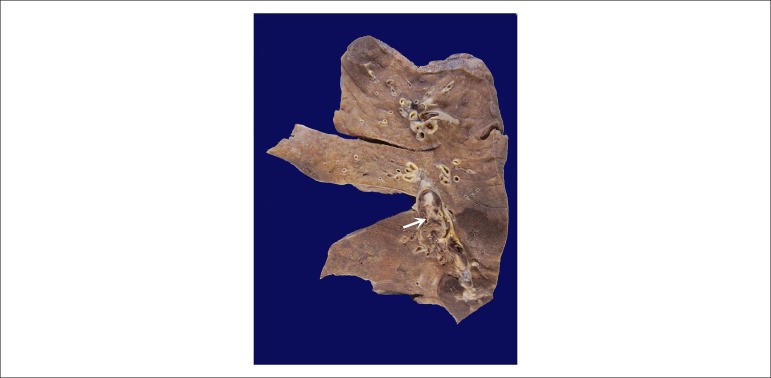
Surface of a lung cut with thrombus-plunger in hilar artery (arrow) and purplish-red areas at the base.

**Figure 9:**
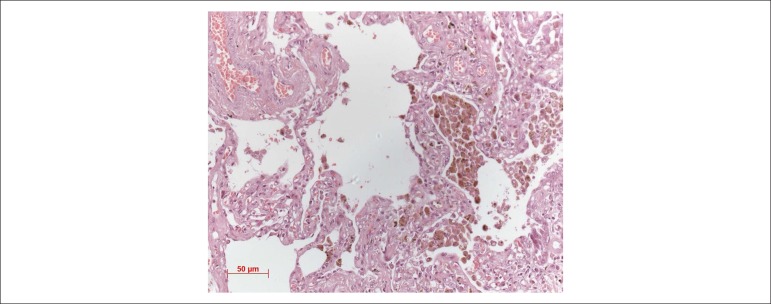
Lung photomicrograph showing histiocytes with hemosiderotic pigment in alveolar lumen (cells of the cardiac defect). Hematoxylin-eosin staining, lens with increase = 5X.

**Figure 10:**
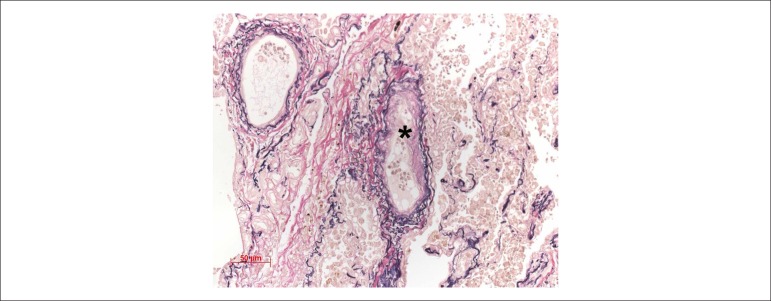
Photomicrography of pulmonary venules with fibrotic lesions in the tunica intima (asterisk). Miller elastin staining, 20X macro lens

**Figure 11:**
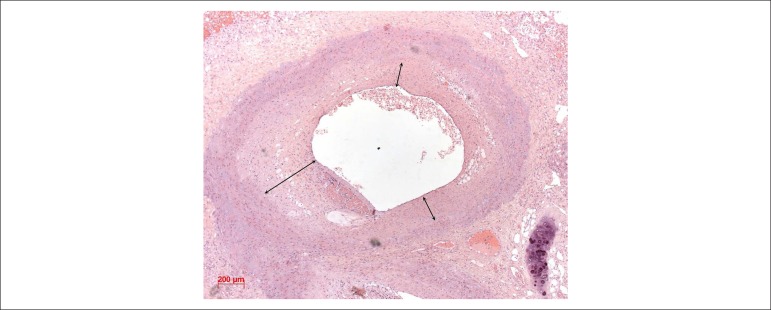
Photomicrograph of a muscular pulmonary artery showing tunica media hypertrophy and proliferative lesion of the tunica intima, concentric (double arrows). Hematoxylin-eosin staining, 5X macro lens.

The exam of the heart showed increased weight (470 g) as well as moderate to significant increase in volume of both atria. The mitral valve showed a lesion characterized by fusion of commissures and marked calcification of the cusps, compatible with rheumatic disease sequelae (Figures 6 and 7). The other valves suffered no significant morphological alterations. The left ventricle had its volume unchanged. There was hemothorax on the left (about 1000 ml). The lungs showed macroscopically thromboembolic vessels in hilar vessels, in addition to purplish-red areas with a firmer consistency in the lower lobes (Figure 8). Histological exam showed recent pulmonary infarctions, organized thrombi in pulmonary arteries, and signs of chronic passive congestion (Figure 9). The wall of the pulmonary veins had thickened, with intimal fibrosis (Figure 10) and hypertrophy of the tunica media of the arteries and arterioles (Figure 11).

In the other organs there were signs compatible with shock, such as, for instance, recent centrilobular hepatic necrosis, acute renal tubular necrosis, and small sub-endocardial infarcts on ventricular walls.

**Anatomopathological diagnoses**: rheumatic heart disease with mitral valve sequelae (calcified mitral stenosis); chronic passive pulmonary congestion with signs of passive pulmonary hypertension; hemothorax on the left without a defined causal factor.

**Cause of death**: Pulmonary thromboembolism (**Prof. Dr. Vera Demarchi Aiello**)

## Comments

The involvement of the mitral valve in this case is typical of rheumatic disease sequelae, and the heart showed signs of de-compensation, such as marked dilation of the atria. Signals of terminal shock were found in the various organs.

The involvement of only the mitral valve is common in rheumatic disease, and it can be found in over 50% of the cases of chronic rheumatic diseases as double dysfunction (stenosis and insufficiency) or just insufficiency.^21^

No lesions were found in arterial or venous thoracic vessels able to explain hemothorax.

The pulmonary situation included recent aspects, such as thromboembolism of hilar vessels and pulmonary infarcts, with other chronic ones, characterized by long-term chronic passive congestion. Passive congestion ends up by causing passive pulmonary hypertension, which starts in the venous territory. The case under discussion presented lesions in venules characteristic of this type of impairment. In the last classification of pulmonary hypertension, this group (of hypertension secondary to lesions of the left heart) is known as pulmonary hypertension group 2.^22^ In addition to the lesions in the cardiac valves on the left side, myocardial diseases can also evolve chronically with secondary pulmonary hypertensive involvement. Individuals thus affected can have a troubled evolution in the postoperative period of valve surgery, or that of a cardiac transplantation, when this is a therapeutic option. A recent study by our laboratory revealed that venous lesions of pulmonary hypertension group 2 are frequent and that the appearance of phosphodiesterase 5 in pulmonary vessels of those patients is greater than in the normal ones.^23^ (**Prof. Dr. Vera Demarchi Aiello**)
